# T207. A REVIEW OF PREDICTORS OF RESPONSIVENESS TO CBT FOR PSYCHOSIS

**DOI:** 10.1093/schbul/sby016.483

**Published:** 2018-04-01

**Authors:** Ana Elisa Farias de Sousa, Martin Lepage, Mathieu Brodeur

**Affiliations:** McGill University

## Abstract

**Background:**

Pharmacological and psychological intervention combined are proved to be more effective for treating psychosis than pharmacological treatment alone. Cognitive Behavioral Therapy for psychosis (CBTp) has been empirically supported as conjoint treatment providing a significant improvement in positive and negatives symptoms, and functional outcomes for psychosis. However, rates of patient discontinuation in CBTp and occasional lack of improvement in symptoms show it is important to refine the identification of the individual characteristics related to better response to CBTp. This literature review aims to accomplish a comprehensive analysis of the evidence-based studies that have searched predictors in the last decades, focusing on individual factors that directly predicts responsiveness to CBTp, rather than therapist or treatment factors. The scope of knowledge gathered here intends to guide practical application of CBTp to people with psychosis that can benefit more from this intervention. Adaptations to improve the effectiveness of CBTp and gaps to be addressed in further research are also considered.

**Methods:**

Thirty (30) studies (18 RCT) were included to determine which characteristics are relevant for a distinctive response to CBTp in people with schizophrenia and other psychotic disorders. The word “predictor” was used to discriminate pertinent studies. Articles were included if they reported in a population within a Psychosis Spectrum Disorder; reported on CBT or derived intervention; reported on individual predictors of outcome in CBT or derived therapy. Articles that reported on a high-risk psychosis population or on comorbidities with psychosis; reported non-individual predictors; were case studies or literature reviews; had a small sample; and had mixed interventions and did not report results specific to CBT were not included.

**Results:**

Studies have shown divergences in methodology, focus on different domains and time-points of disorders outcome and great heterogeneity in results. There is strong evidence that greater clinical and cognitive insight, cognitive flexibility, greater positive symptom severity and less pronounced negative symptoms at baseline, shorter duration of psychosis, a greater number of hospitalization in the previous five years and pre-therapy coping styles can predict better outcome in CBTp, although their significance has varied between studies. While impairment in verbal memory was related to a shortage of improvement in symptoms and a greater likelihood to abandon of treatment before completion, most studies did not find neurocognitive functioning to be a predictor of outcome in CBTp.

**Discussion:**

Further investigation is needed to determine the extent and validity of these predictors in different populations within the scope of psychosis. Professionals can benefit from the gathered knowledge, using these findings to better target CBTp and to focus early stages of intervention on developing patient’s abilities such as cognitive flexibility and insight, working memory, coping skills and clinical awareness in order to improve their receptiveness to therapy and successful outcome.

Future research should aim to replicate findings with larger and more diagnosis-comprehensive samples to enable generalization of the present results. Aspects such as personality traits, metacognition and sociodemographic characteristics require more thorough investigation to confirm their predictive value before being taken into consideration when selecting patient suitability to CBTp and similar interventions.

